# Does Access to Research Trials Contribute to Improved Dementia Diagnosis for Service Users at NHS Memory Clinics?

**DOI:** 10.1192/bjo.2026.11738

**Published:** 2026-06-30

**Authors:** Margaret Butler, Vanessa Mabiala, Ramin Nilforooshan

**Affiliations:** Surrey and Borders NHS Trust, Leatherhead, Surrey, United Kingdom

## Abstract

**Aims:**

Alzheimer’s disease (AD) diagnosis is shifting from clinical diagnosis to a biomarker-guided pathway. An estimated 60% of people with dementia receive a diagnosis and 25-30% are misdiagnosed showing need for optimized diagnostic approaches. Blood-based biomarkers (BBBMs) may identify AD before symptoms and differentiate between AD from other dementia subtypes, with similar accuracy to CSF and PET biomarkers. This paper examines whether access to clinical trials can contribute to improved dementia diagnosis for service users at a National Health Service memory assessment service.

**Methods:**

An audit was registered with the local NHS Trust. Data was retrospectively collected from 69 participants screened for dementia drug trials by the Research & Development service between May 2024 and October 2025, healthy volunteers, people with Mild Cognitive Impairment (MCI) due to AD and Mild AD. Medical records from memory clinic assessment and trial data were anonymised and analysed.

**Results:**

Of 69 participants, 6% were healthy volunteers. At memory clinics, 93% underwent Addenbrooke’s Cognitive Examination-III and 80% brain imaging, mostly MRI (42%), 28% CT, 7% MRI and FDG-PET. Following memory service assessment, AD was lead diagnosis (42%), MCI (30%), 7% no diagnosis.

During trial screening, 75% of participants received a plasma p-tau test (28% had positive results). 52% had brain imaging, mostly MRI head plus amyloid PET (42%). 28% had positive amyloid PET scans and 13% had positive tau-PET scans.

Following screening, 83% of participants retained their initial diagnosis. 10% received a new diagnosis while, 7% of participants await reassessment.

**Conclusion:**

17% of study participants’ diagnoses changed course in clinical trials, possibly due to access to additional investigations. This paper highlights the potential benefit of NHS participation in research and a need for serial assessment and reversal of diagnosis pathways in dementia and for further research in this area.

